# Dance Communication Affects Consistency, but Not Breadth, of Resource Use in Pollen-Foraging Honey Bees

**DOI:** 10.1371/journal.pone.0107527

**Published:** 2014-10-01

**Authors:** Matina Donaldson-Matasci, Anna Dornhaus

**Affiliations:** 1 Department of Biology, Harvey Mudd College, Claremont, California, United States of America; 2 Department of Ecology & Evolutionary Biology, University of Arizona, Tucson, Arizona, United States of America; University of Guelph, Canada

## Abstract

In groups of cooperatively foraging individuals, communication may improve the group’s performance by directing foraging effort to where it is most useful. Honey bees (*Apis mellifera*) use a specialized dance to communicate the location of floral resources. Because honey bees dance longer for more rewarding resources, communication may shift the colony’s foraging effort towards higher quality resources, and thus narrow the spectrum of resource types used. To test the hypothesis that dance communication changes how much honey bee colonies specialize on particular resources, we manipulated their ability to communicate location, and assessed the relative abundance of different pollen taxa they collected. This was repeated across five natural habitats that differed in floral species richness and spatial distribution. Contrary to expectation, impairing communication did not change the number or diversity of pollen (resource) types used by individual colonies per day. However, colonies with intact dance communication were more consistent in their resource use, while those with impaired communication were more likely to collect rare, novel pollen types. This suggests that communication plays an important role in shaping how much colonies invest in exploring new resources versus exploiting known ones. Furthermore, colonies that did more exploration also tended to collect less pollen overall, but only in environments with greater floral abundance per patch. In such environments, the ability to effectively exploit highly rewarding resources may be especially important–and dance communication may help colonies do just that. This could help explain how communication benefits honey bee colonies, and also why it does so only under certain environmental conditions.

## Introduction

For animals living in social groups, collecting food is often a collaborative venture. Individuals foraging in groups may benefit from sharing information with one another, because then they need invest less effort into gathering information and can devote more effort into gathering resources [1], [2]. In eusocial insects, like honey bees and ants, flexible division of labor may allow some individuals to focus on collecting information about new and previously rewarding resources, while others focus on exploitation of known resources [3], [4]. Communication is thought to play a key role in regulating this process [5], [6]. For example, honey bee foragers fan out over the landscape from their hive, searching for flowers rich in pollen or nectar. Those that are successful may choose to recruit more foragers to the same patch, using the famous “dance language” to communicate the flowers’ location [7]. Similarly, many ant species use pheromone trails to recruit helpers to particularly rewarding food sources. In both of these cases, it is thought that communication about high-quality resources creates a feedback loop: each new forager recruited may in turn recruit more, until overexploitation reduces the rate at which rewards can be collected [8], [9]. This feedback loop leads to a preferential buildup of foragers at higher quality resources [10]–[12]. Although the results of this dynamic process have not been well quantified in nature, it is thought that for many trail-laying ants the result is a collective decision to exploit only the best resource, while honey bees are able to maintain some level of foraging effort even on lower-quality resources [12]–[16].

How honey bees use the dance language to communicate is now quite well understood [17]; however, exactly why they do so is still something of a mystery [18], [19]. How does communicating about where to find food resources actually benefit a colony? One important hypothesis is that communication makes food collection more efficient, because it tends to change the allocation of foraging effort across resources according to resource quality [10], [20]. In support of this, honey bees that follow dances have been shown to collect more nectar than those that search for flowers on their own [21]. Furthermore, the ability to effectively communicate location information via the dance increases the amount of nectar an entire colony can collect, at least in some environments [18], [22]. This effect is strongest in environments where there are many species of flowers in bloom [23]. One potential explanation is that when many species are in bloom, flower patches vary widely in the quality and quantity of nectar they provide–making it especially valuable for the colony to be able to concentrate more of their foraging effort on the best ones.

Despite mounting evidence that dance communication helps honey bee colonies collect more nectar by directing foragers to high-quality nectar sources, little is known about how communication may influence the way colonies forage for pollen. Most research on dance communication in honey bees has been done on nectar foraging, typically with artificial nectar sources whose quality can easily be manipulated by changing the sugar concentration [7], [10], [24]. To study how colonies forage on natural resources, which are too numerous to monitor directly, another approach is to decode the dances and create a map of the locations being advertised [18], [25]–[27]. However, this gives information only about the resources that motivated foragers to dance, not all resources being used by the colony. To understand how dance communication affects the way a colony spreads its foraging effort across different natural resources, we need another way to identify those resources. The complex structure of pollen grains gives us a way to recognize different sources; fine structural details of pollen grains can be used to identify the plant species (or higher taxonomic level) they were collected from.

In this article, we test the hypothesis that dance communication changes the way that honey bee colonies allocate their foraging effort across different naturally occurring pollen sources, and examine the effect on foraging success. To do this, we manipulated colonies’ ability to communicate about resource location across five different natural habitats, and looked for changes in the amount and composition of pollen collected. We taxonomically identified the pollen each colony collected to not only determine which floral resource types the colony used, but also estimate the relative foraging effort dedicated to each type. We also directly assessed the floral resources available to the bees in each habitat. This allowed us to relate the floral resources these colonies used to those available to them, and see how communicating about resource location might change that relationship.

We examine the hypothesis that communication helps colonies forage more efficiently by shifting their effort towards more rewarding pollen resources. If this were the case, we would expect to see that colonies with intact communication allocate a greater proportion of their foragers to resources offering higher quantity and/or quality of pollen, in comparison to colonies with impaired communication. Unfortunately, we cannot directly assess this prediction, because it is not clear exactly how honey bees evaluate pollen resources [28]–[31]. Instead, we consider several possible ways that communication might alter the way colonies distribute foragers across pollen resource types. First of all, shifting foraging effort towards better resources could cause floral sources with low pollen rewards to be abandoned altogether, particularly if they are not very abundant. If so, (1) colonies with intact communication might exploit fewer different pollen types each day, compared to those with impaired communication, thus displaying a lower richness of resource types used. Second, even if poor resources are not completely abandoned, colonies with intact communication might still concentrate more of their foraging effort on highly rewarding pollen sources. In this case, (2) colonies with intact communication might show a lower diversity of resource use per day (i.e. a more uneven distribution across types, relying more heavily on just one or a few major types), compared to those with impaired communication. Third, if communication tends to concentrate colony foraging effort on a specific set of resources, (3) we might expect colonies with intact communication to consistently use the same set of pollen types, both across days and colonies, while colonies with impaired communication might be more likely to explore novel pollen types. Finally, if colonies use communication to shift their foraging effort towards more rewarding resources, (4) changes in resource use associated with impaired communication might reduce the quantity of pollen that colonies collect.

## Methods

To see how communication affects resource use in pollen-foraging honey bees, we taxonomically identified the pollen collected by six colonies under two different communication treatments, and compared this to the floral resources available to them at the time. This was repeated in a series of five experiments across different Sonoran desert habitats and seasons, chosen to maximize differences in the floral resources available (see [23] for a report on other aspects of the same set of experiments).

### Communication treatments

To determine how the honey bee dance communication system affects colony-level foraging patterns, we used an established technique to manipulate colonies’ ability to communicate about resource location (as described in [23]; see also [7], [18], [19], [22], [32]–[34]). Under normal conditions, directional information is encoded in the angle of a forager’s dance, where running straight up the vertical comb in the darkness of the hive indicates a floral source in the same direction as the sun. Turning the hive on its side disrupts the reference point for the dance (gravity), so that bees no longer dance in consistent directions, and recruitment is no longer location-specific [32]. However, honey bees can use a directional light source as an alternative reference point, restoring location-specific recruitment [22], [33], [34]. We compare two communication treatments, both in specially constructed hives turned on their sides: (1) *impaired communication*, where no reference point for the dance was available, and (2) *intact communication*, in which a source of directed light provided an alternative reference point for the dance. Previous research using the same hive design has demonstrated that dances occur at the same rate in both communication treatments [22]. However, compared to the intact-communication treatment, dances in the impaired-communication treatment are more often disoriented, with waggle runs in random directions [18], [22], [34], resulting in decreased recruitment to artificial feeders [22], [33]. Furthermore, under some environmental conditions, the impaired-communication treatment also reduces the colony’s nectar collection (this experiment: [23]; a previous experiment using the same hive design: [18]). In each 12-day experiment, six colonies of about 10,000 domestic Italian honey bees (*Apis mellifera ligustica*) were rotated through 3-day treatment blocks with communication either impaired or intact, staggered so that on each day three colonies had intact communication and three colonies had impaired communication (see [23] for detailed experimental schedule). Treatments are thus balanced for colony effects, since each colony experienced both treatments, and for weather effects, because on each day half of the colonies were in each treatment.

### Experiment dates and locations

A series of five experiments were performed from April until October 2010, at four different sites within a 100 km radius of Tucson, Arizona. Experiment 1 was performed in Sonoran desert scrub habitat (at the non-profit Sonoran Arthropod Studies Institute, SASI, in the Tucson Mountains) during late spring (April 21–May 2). Experiment 2 was performed in the same location, but during foresummer drought, which is an exceedingly hot, dry period in early summer characteristic of the Sonoran desert (May 20–June 1). Experiment 3 was performed in mesquite-oak grassland habitat (at the Audobon Society’s Appleton-Whittell Research Ranch, AWRR) also during the foresummer drought (June 18–30). Experiment 4 was performed in the mouth of a riparian canyon (at the University of Arizona’s Santa Rita Experimental Range headquarters in Florida Canyon, SRER) during the monsoon season (August 9–20). Experiment 5 was performed again in Sonoran desert scrub habitat (at the University of Arizona’s Desert Station in the Tucson Mountains, UADS) in the fall (October 9–20). Because all field experiments took place on land owned by the university or by non-profit organizations, research permits were not required.

### Floral resource surveys

We surveyed the plant species in bloom during each experimental period, and assessed the floral abundance and spatial distribution of each species using censored T-square sampling [35], [36]. For each experimental site, we chose 14–18 random sampling points within a 0.5 km distance of the hive, and measured the distance to the nearest blooming plant of each species, the distance to the next blooming plant of the same species, and estimated the number of flowers on each plant. From these measurements, we first estimated the patch density 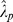, that is, how much area must be searched, on average, before finding the first flower. Second, we estimated how clustered those resources are in space as the number of flowers per patch 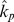, that is, once the first flower has been found, how many more are likely to be found nearby (for details of these calculations, see [23]).

To estimate species richness, while correcting for slight differences in sampling effort between experiments (14–18 sampling points per experiment), we fitted a Monod species accumulation curve to each experiment, and standardized at 18 sampling points using the R packages vegan and mmSAR [37]–[39]. To estimate the diversity of floral resources available, we calculated Shannon’s diversity index 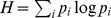, where 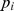 is the relative abundance of flowers estimated for each species, calculated from our estimate of absolute abundance 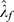 (see [23]). The Shannon index is a common measure of ecological diversity, which is highest when there are many different species and those species are found in approximately equal proportions [40].

### Pollen collection and identification

We collected pollen from honey bee colonies by mounting pollen traps over their hive entrances; entering foragers were forced to squeeze through a plastic grate, scraping the pollen loads from their legs. Pollen was collected from the pollen traps daily, weighed, labeled and frozen for later identification. Each day, just two colonies were fitted with a pollen trap: one in each communication treatment (impaired and intact). Each colony had the pollen trap once per treatment block, i.e. every third day [23]. This resulted in a total of 106 samples: 2 colonies per day, 12 days per experiment (except Experiments 2 and 3, which lasted an extra day), and 5 experiments would be 124 samples, but some samples contained no pollen.

For each of these samples, a subsample of approximately 30–40 corbicular loads were counted out (0.2 mL measured by displacement) and thoroughly mixed by softening for 1 hour in distilled water with a small amount of surfactant, stirring and then vortexing each sample. The samples were centrifuged and the resulting pellets were given to an expert palynologist (Owen Davis, Department of Geosciences, University of Arizona) for identification. Each pollen sample was prepared with acetolysis, stained with fuchsin, and mounted for examination by light microscopy with a Zeiss Universal Fluoroscope 61110 at 250–450x. This technique brings out unique features of the pollen exine (outer wall) and allows pollen from different plant taxa to be distinguished; identification is generally possible only to genus or family, but sometimes to species. For each sample, a count of approximately 500 pollen grains was made over a transect from left to right across the center of the cover slip, with additional transects at ¼ of the cover slip above and below that if necessary. Each pollen grain was identified by comparing it to an extensive reference library of Sonoran desert pollen types, providing an estimate of how many different taxa were collected by that colony on that day, and of the relative abundance of each taxon in the sample [41]. The Shannon diversity index (H) was calculated for each sample based on the proportions of different pollen types observed. For extremely low-diversity samples (e.g. more than 95% of pollen grains of a single type), the ratio between the major type and all other types was determined in a first count of 500 pollen grains as described above, and then the relative abundance among the rest was determined in an additional count of 100 pollen grains of just the other types combined.

### Statistical analyses

All statistical analyses were performed using either linear or generalized linear mixed-effects models, depending on the nature of the response variable. Linear mixed-effects models were used to analyze the effects of communication treatment on both (1) the number of pollen types and (2) the Shannon diversity of pollen types, with communication treatment and either flower species richness or diversity as fixed effects, and date and colony (within experiment) as random effects. To look at (3) the consistency of pollen use over time, we looked at the effects of communication treatment on the proportion of novel types, that is, out of all pollen types collected by a particular colony on a given day, how many of those were never observed in any other sample. The proportion of novel types was modeled using mixed-effects logistic regression (i.e. a binomial generalized linear mixed-effects model), again with communication treatment and flower species richness as fixed effects, and date and colony (within experiment) as random effects.

In a previous report on this experiment, we analyzed the combined effects of communication treatment and habitat on the amount of pollen collected [23]. To do this, we transformed pollen weight into a binary response variable by asking whether a colony collected more pollen than the other colony fitted with a pollen trap on the same day. This method has the advantage of controlling for extreme changes in foraging success from day to day, caused by differences in foraging conditions. We then modeled the transformed variable using mixed-effects logistic regression with communication treatment and three characteristics of the floral resource distribution as fixed effects, as well as the interaction between communication treatment and each floral resource variable (species richness, patch density, and flowers per patch). We included colony as a random effect, but not date, because by comparing only within days we control for date. For consistency with the other models presented in this article, we have reanalyzed these data and here present the model with colony within experiment as a random effect, to account for the possibility that colony characteristics changed over the course of the series of five experiments; the results, however, were qualitatively the same.

Finally, to look at (4) the effects of resource use on the amount of pollen collected, we asked whether the differences we found in the consistency of pollen use between communication treatments could explain the observed effect of communication treatment on pollen weight across habitats. To do this, we constructed two mixed-effects logistic regression models similar to the one described above. In the first model, we replaced communication treatment with the number of novel pollen types a colony collected. In the second model, we included both communication treatment and the number of novel pollen types as fixed effects, along with their interactions with each of the three floral resource variables.

All models were implemented using the lme4 package in R [42], [43]. To determine whether a specific fixed effect was important, we used a likelihood ratio test (LR test) comparing nested models with and without that term.

## Results

**Figure 1:**
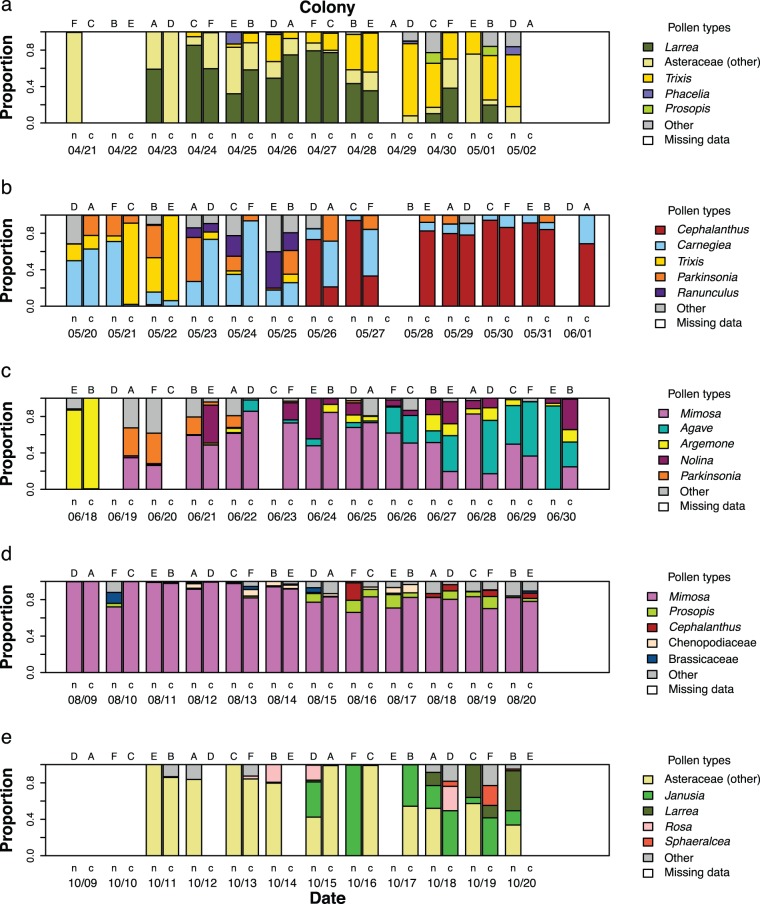
Pollen types used by honey bee colonies. For each colony (letters A–F, top axis) on each day (bottom axis), we show the type composition of the pollen collected, highlighting the five most common pollen types in each experiment. On each day, there is one colony with impaired communication (labeled n) and one colony with intact communication (labeled c). For some samples, no pollen was collected (missing data). Data for Experiments 1–5 are shown in panels a–e.

Most colonies collected between 3 and 6 different pollen types per day (median 5 types, range 1–14 types), but typically concentrated on just one or two major types (mean (± SE) frequency of most prevalent type per sample = 73±2%). The range of pollen resources used differed dramatically among experiments, and often changed over time within experiments. For example, in the first half of Experiment 2, colonies mainly collected pollen from saguaro cacti (*Carnegiea*), threefold (*Trixis*), and palo verde trees (*Parkinsonia*), while buttonbush (*Cephalanthus*) became the most heavily used pollen source in the second half of the experiment (see Figure 1b). In contrast, *Mimosa* (likely the abundantly flowering velvetpod mimosa, *Mimosa dysocarpa*) was the major resource for all colonies throughout Experiment 4 (see Figure 1d).

Honey bee colonies did not utilize all the floral resources we observed in bloom (13%–65%, depending on habitat). For example, although desert ironwood (*Olneya tesota*) was blooming profusely during Experiment 2, no pollen was collected from this species. In addition, bees collected pollen from a number of plant taxa that we did not observe in our floral surveys, such as *Agave* and *Nolina* (probably desert spoon, *Dasylirion wheeleri*) in Experiment 3. For full lists of all floral species observed and all pollen types identified in each of the five experiments, see Data S1 and Data S2 respectively.

### (1) Number of pollen types per colony per day

**Figure 2:**
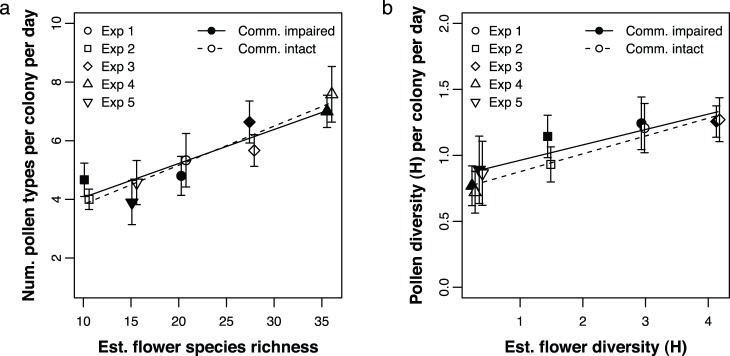
Pollen type richness and diversity per sample. (a) The number of pollen types collected by each colony on each day, as a function of communication treatment (intact or impaired) and the number of floral resources available (as estimated by floral surveys in Experiments 1–5). (b) The Shannon diversity of pollen types collected by each colony on each day, as a function of communication treatment and the Shannon diversity of floral resources available. Lines show predicted values from the linear mixed-effects model (see text for details); error bars are standard errors. The *x* axis values for the treatment with intact communication are slightly offset, so that the error bars do not overlap.

If communication tends to restrict a colony’s foraging effort to specific pollen types, colonies with impaired communication might collect pollen from a wider range of resources. To test this prediction, we looked at the effect of floral species richness and communication treatment on the number of types each colony collected. We found that, comparing across experiments, the number of different pollen types used by each colony per day increased significantly with the number of different plant species in bloom (LR test: χ^2^ = 19.90, *p* = 8×10^−6^; see Figure 2a). However, contrary to prediction, there was no effect of communication treatment (LR test: χ^2^ = 0.013, *p* = 0.90).

### (2) Diversity of pollen types per colony per day

If communication tends to concentrate a colony’s foraging effort more heavily on specific pollen resources, colonies with impaired communication might collect pollen from a greater diversity of resource types. As shown above, the number of different resource types does not seem to be affected by communication treatment. However, it is still possible that the proportions of the different types – the second key component of diversity – could be affected. In particular, colonies with impaired communication might be more likely to collect relatively even proportions of different types (high diversity), rather than skewing heavily towards certain types (low diversity). To test this prediction, we looked at the effect of floral species diversity and communication treatment on the diversity of pollen types each colony collected. We found that the diversity of pollen types used by each colony on each day increased significantly as resource diversity increased (LR test: χ^2^ = 9.79, *p* = 0.002; see Figure 2b). Note that this relationship between floral resource diversity and pollen diversity is not simply driven by the relationship we observed between floral species richness and the number of pollen types: the two experiments with the highest observed floral species richness (Experiments 3 and 4) had the highest and lowest floral resource diversity, respectively. Still, as above, and contrary to prediction, there was no effect of communication treatment (LR test: χ^2^ = 0.571, *p* = 0.45).

### (3) Consistency across colonies and days

**Figure 3:**
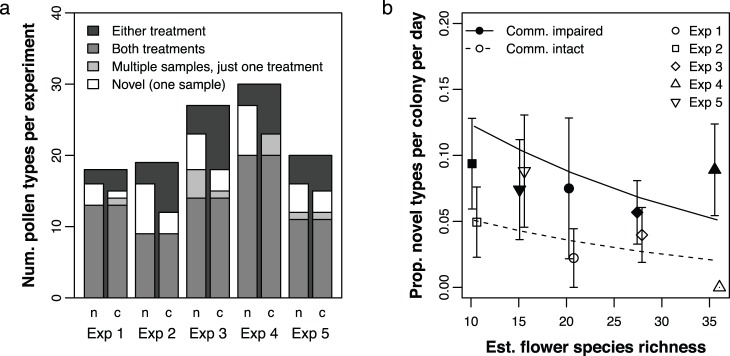
Novel pollen types. (a) The total number of pollen types observed in each experiment, categorized according to whether they were collected by colonies with communication intact (bars labeled ‘c’) and/or with communication impaired (bars labeled ‘n’). Pollen types collected in just one of the two treatments are further subcategorized according to whether they were collected by just one colony on one day (novel types) or by multiple colonies and/or on multiple days. (b) For each colony on each day, the proportion of novel pollen types is calculated as the number of pollen types found only in that sample divided by the total number of types in that sample. This is plotted as a function of communication treatment and the number of floral resources available. Lines show predicted values from the logistic regression (see text for details); error bars are standard errors. The *x* axis values for the treatment with intact communication are slightly offset, so that the error bars do not overlap.

If communication tends to focus a colony’s foraging effort on specific pollen resources, colonies with impaired communication might utilize a broader range of resource types. If this is true, we would expect colonies with intact communication to use fewer resource types overall, i.e. to share more resource types both across colonies and across days. Conversely, we would expect colonies with impaired communication to show less overlap in the pollen sources they use, both across colonies and across days. To assess this, we pooled all samples (different colonies and different days) within each experiment and communication treatment, for a total of 10 sample pools (5 experiments × 2 treatments). We then calculated the total number of distinct pollen types observed in each sample pool. Within each of the five experiments, we compared the two sample pools with different communication treatments. We observed that in each case the sample pool with intact communication contained fewer pollen types overall than did the sample pool with impaired communication (see Figure 3a). Much of this discrepancy can be explained by a difference in the number of novel pollen types collected, i.e. pollen types that were just collected by one colony on one day, within each experiment.

Since we did not find a significant difference in the number of pollen types collected each day between colonies with intact and impaired communication, the difference in overall number of types suggests that colonies with intact communication tended to repeatedly use the same set of pollen resource types (both between colonies and on different days), while colonies with impaired communication were more likely to collect novel pollen types. To confirm this difference in consistency of pollen resource use, we looked at the *proportion of novel types*, that is, for each colony what fraction of the pollen types they collected each day were novel (not collected before or again by any colony). We asked whether the proportion of novel types depended on communication treatment and the number of floral resource types available. We found that colonies with communication intact were significantly less likely to collect novel pollen types than those with communication impaired (LR test: χ^2^ = 7.58, *p* = 0.006; see Figure 3b). Flower species richness did not have a significant effect on the proportion of novel types (LR test: χ^2^ = 2.94, *p* = 0.09).

### (4) Impact of shifts in pollen resource use

**Figure 4:**
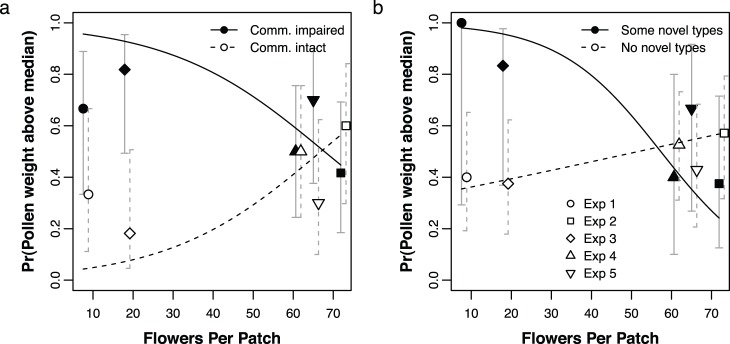
Amount of pollen collected. Each day, we weighed the pollen that two colonies collected; we asked what affected how often one colony collected more pollen than the other in its pair. (a) As previously reported, we found that the effects of communication treatment (impaired or intact) on amount of pollen collected depend on habitat; specifically, intact communication is only beneficial when the number of flowers per patch is high [23]. (b) Here we show that the collection of novel pollen types also affects how much pollen is collected, again depending on habitat. In particular, collecting novel pollen types is more detrimental when the number of flowers per patch is higher. Lines show predicted values from the logistic regressions (see text). Error bars are 95% confidence intervals calculated for the observed proportions. *x* axis values are slightly offset by treatment (in a: colonies in treatment with intact communication) or grouping (in b: colonies that collected no novel types), so that the error bars do not overlap.

If communication tends to shift a colony’s foraging effort towards more rewarding resources, colonies with impaired communication might collect smaller quantities of pollen. However, a previous analysis of the benefits of communication on the same set of experiments found that intact communication did not always increase the amount of pollen collected [23]. For completeness, we present that analysis again here (see Figure 4a). We found that across all experiments colonies with communication intact typically collected less pollen than those with communication impaired (LR test, χ^2^ = 8.09, *p* = 0.004). However, as reported previously, the magnitude of this effect diminished as the number of flowers per patch increased (LR test: χ^2^ = 11.2, *p* = 0.0008), so that in Experiment 2 (an environment with flowering trees such as *Parkinsonia*), colonies with communication intact collected more pollen than those with communication impaired.

To determine whether the shifts in pollen resource use we observed could help explain these patterns, we asked whether the number of novel pollen types collected by one colony on a given day could predict how much pollen the colony collected that day. The number of novel types had no overall effect on the amount of pollen collected (LR test: χ^2^ = 0.03, *p* = 0.96). However, in habitats with more flowers per patch, colonies that collected more novel types also collected less pollen (LR test: χ^2^ = 8.53, *p* = 0.003; see Figure 4b).

In certain habitats, consistent foraging on particular resource types thus seems to correlate with increased foraging success. Since colonies with communication intact forage more consistently (rather than collecting novel pollen types each day), could this increased consistency explain why the benefits of communication are higher in those same habitats? We were not able to directly test this here. However, we wanted to be certain that the number of novel types collected, i.e. the consistency of resource use, had a direct effect on pollen foraging success, and that this apparent relationship was not a result of both being affected by communication treatment. To test this, we asked whether the amount of pollen a colony collected on a given day was better explained by both the interaction between communication treatment and flowers per patch and the interaction between number of novel types and flowers per patch, or either interaction alone. We found that both interaction effects contributed significantly to the model, even when the other was present (communication treatment × number of flowers per patch, LR test: χ^2^ = 7.37, *p* = 0.006; number of novel types × number of flowers per patch, LR test: χ^2^ = 6.72, *p* = 0.01). This shows that the number of novel pollen types collected does affect pollen foraging success (in some environments), and that this is not a side effect of communication treatment affecting both number of novel pollen types and pollen foraging success. The higher success of colonies that forage consistently is unlikely to be just a result of omitting novel types, because the novel types themselves generally comprise only a very small proportion of all pollen a colony collects (mean 1.03%, range 0.003%–10.9%). In summary, we conclude that colonies foraging consistently, focusing on the same resource types day after day, are likely to be more successful pollen foragers in habitats with many flowers per patch. Communication may be one mechanism that helps colonies achieve this.

## Discussion

Previous research on the honey bee dance has found that, despite its conspicuous role in communicating about food resources, it does not always seem to improve foraging success [18], [21], [22]. This begs the question: when is dance communication useful, and why did it evolve? Theoretical and empirical studies have suggested that one important function of dancing is to improve the group’s foraging efficiency by focusing foraging effort on the most rewarding resources [10], [11], [44]. Therefore, communication might be particularly useful in environments with many resource types that vary in quality [23], [45], [46]. This hypothesis about how dance communication benefits colony foraging also predicts that, if communication is impaired, colonies will be less selective about which resource types they use–and that this change in resource use may be detrimental to foraging success, at least in some habitats.

We tested these key predictions here. We found that, indeed, impairing communication affected the colony-level pattern of resource use, but the changes were quite subtle. Impairing communication did not affect the number or diversity of pollen types that colonies collected each day, as would have been expected if communication focused all or most of a colony’s foraging effort on a few highly productive pollen resources. Instead, we found that colonies with impaired communication more often brought in rare, novel pollen types, rather than consistently foraging on similar resource types from day to day. This suggests that communication enabled colonies to maintain their foraging effort on previously discovered, rewarding food sources, while exploring fewer new sources each day. We also found that colonies which collected from more novel resource types were less successful, i.e. collected less pollen overall, in habitats with many flowers per patch. Together, these observations may partially explain our previous finding that the benefits of communication for pollen collection increase with number of flowers per patch [23].

How might novel resource use relate to pollen foraging success? One possibility is that colonies that spend a lot of time and energy collecting novel resources might not be able to collect as much pollen from highly productive pollen sources. In agreement with this, in some environments using novel resources is associated with smaller amounts of pollen gathered overall. However, that relationship does not seem to be a result of colonies collecting less of any particular major pollen type, such as *Mimosa* in Experiment 4. This suggests that the treatment effects we see may not be due to a strong redistribution of foragers across different floral resource types. However, the treatment may affect the way foragers are distributed across different patches of the same flower type. In habitats with few flowers per patch, a colony may need to exploit many different patches in order to collect enough pollen. In such situations, scouting for new resource patches – sometimes encountering rare, novel types – may be quite useful, while the ability to thoroughly exploit known resource patches may be less critical. However, in habitats with many flowers per patch, a colony needs to discover just a few rewarding patches, and may be able to improve foraging success by using dance communication to consistently focus on the same patches, rather than searching for new ones.

A previous study on the effects of honey bee worker genetic diversity on pollen collection also shows a connection between resource use and foraging success [47]. In that study, colonies with lower genetic diversity collected smaller quantities of pollen overall, and that pollen came from a greater number of different resource types. Furthermore, the additional pollen types were generally observed in very low quantities, like the novel pollen types in our experiment. Because foragers in colonies with low genetic diversity are less likely to dance [48], such colonies may depend less on recruitment and more on independently searching foragers. Along with our own results, these studies support the idea that an impaired dance communication system may lead colonies to scout for new resources more intensively and exploit known resources less thoroughly.

Another reason that colonies in the impaired communication treatment might discover more new food sources is that bees following disoriented dances may search in the wrong direction, and sometimes find novel resources as “lost recruits” [3]. Earlier research using the same hive design looked at how communication treatment affected recruitment rates to an artificial feeder, while also measuring the rate of arrival at an unrewarding control feeder in the opposite direction [33]. Few bees arrived at the control feeder regardless of treatment, suggesting that disoriented dances do not increase the rate at which new resources are discovered via lost recruits. Coupled with previous research [47] showing that colonies with low genetic diversity (and thus reduced dance rates) also tend to explore a greater variety of resources, this suggests that the increased use of novel resources we observed is a robust response to lack of informative dances, not an artifact of misinformation in disoriented dances.

Animals foraging on multiple, variable resources must strike a delicate balance between exploring the potential resources that are available, and exploiting the actual resources they already know. The optimal balance depends on the environment, e.g. the abundance and variability of the resources [49]. In collectively foraging groups, communication may play an important role in regulating that balance [6]. Our empirical results suggest that dance communication does indeed reduce the amount of effort a honey bee colony devotes to the exploration of resources, and that in some environments – when each patch contains many flowers – this also allows for more efficient exploitation of resources. The remarkable dance communication system of honey bees may thus have evolved as a way to regulate the group’s exploratory effort in situations where exploitation of a few highly rewarding resources is particularly valuable.

## Supporting Information

Data S1 **Flower species observed in floral surveys for each of the five experiments.** For each experiment, a list of all flower species observed in bloom during the floral survey is recorded, including the common name, the species name, and the family.
(XLSX)

Data S2 **Plant taxa identified in pollen samples collected during each of the five experiments.** In this table of all pollen types observed (rows) and all five experiments (columns), an “X” indicates the presence of a particular pollen type in at least one sample from a particular experiment. Blank indicates that no samples from that experiment contained that pollen type. Note that the taxonomic level for pollen types may be species, genus, or even family, depending on how similar pollen grains in that group are.
(XLSX)
